# Clustering of chronic disease risks among people accessing community mental health services

**DOI:** 10.1016/j.pmedr.2022.101870

**Published:** 2022-06-27

**Authors:** Casey Regan, Caitlin Fehily, Elizabeth Campbell, Jenny Bowman, Jack Faulkner, Christopher Oldmeadow, Kate Bartlem

**Affiliations:** a School of Psychological Sciences, The University of Newcastle, Callaghan, NSW 2308, Australia; b Hunter New England Population Health, Locked Bag 10, Wallsend, NSW 2287, Australia; c The Australian Preventive Partnership Centre (TAPPC), Sax Institute, Ultimo, NSW, Australia; d Hunter Medical Research Institute, Kookaburra Circuit, New Lambton Heights, NSW 2305, Australia; e School of Medicine and Public Health, The University of Newcastle, Callaghan, NSW 2308, Australia

**Keywords:** Clustering, Chronic disease risks, Tobacco smoking, Alcohol consumption, Fruit and vegetable intake, Physical activity, Body Mass Index, Mental health conditions, Community mental health services

## Abstract

This study identified clusters of chronic disease risks and explored associations between clusters and demographic characteristics and mental health conditions, among people accessing community mental health services. Data from a cross-sectional telephone survey of Australian mental health consumers (n = 567) were analysed. Clusters were identified based on tobacco smoking (53.5%), harmful chronic alcohol consumption (20.1%), harmful acute alcohol consumption (43.5%), inadequate fruit and vegetable intake (66.0%), inadequate physical activity (75.5%), inadequate strength activity (81.8%), and high body mass index (BMI) (67.9%), using latent class analysis. Multinomial logistic regression examined associations between cluster membership and participant characteristics. Three groups were identified: Cluster 1 (19.05%) had < 0.5 probabilities for most risks; Cluster 2 (34.04%) had high probabilities of all risks, particularly tobacco smoking and both types of harmful alcohol consumption; and Cluster 3 (46.91%) had high probabilities of both inadequate physical and strength activity, inadequate fruit and vegetable intake, and high BMI. Compared to Cluster 1 membership, participants with higher education were less likely to be in either Cluster 2 or 3, females or those over 55 were more likely to be in Cluster 3, those with a substance use disorder were more likely to be in Cluster 2, and those with a personality disorder were less likely to be in Cluster 3. The clustering patterns reinforce the importance of addressing multiple chronic disease risks for people with a mental health condition**.** Preventive care interventions targeting clusters of risks may help reduce the burden of chronic disease among this high-risk population.

## Highlights

• Seven chronic disease risks were analysed among community mental health consumers.
• Latent class analysis identified three clusters of chronic disease risks.
• Education level, gender, age and diagnosis were associated with cluster allocation.
• Clustering patterns reinforce the importance of addressing multiple risks.
• Findings can be used to assist the development of multi-risk interventions.

## 1 Introduction

People with a mental health condition are more likely to have modifiable chronic disease risks relative to the general population internationally (Lê Cook et al., 2014, Di Florio et al., 2014, Teasdale et al., 2019, Vancampfort et al., 2017) and in Australia (Bartlem et al., 2015, Australian Bureau of Statistics, 2018), including tobacco smoking, harmful alcohol consumption, inadequate fruit and vegetable intake, inadequate physical activity, and overweight and obesity. The presence of any mental health condition is associated with elevated rates of each risk (Firth et al., 2019), and more severe mental health conditions, such as bipolar disorder and schizophrenia, are most strongly associated with risks (Jackson et al., 2015, Di Florio et al., 2014, Teasdale et al., 2019, Firth et al., 2018, Vancampfort et al., 2017) relative to other conditions. Whilst the presence of multiple risks is common among the general population (Meader et al., 2016, Noble et al., 2015), the likelihood of multiple risks is even higher for people with a mental health condition (Bartlem et al., 2021, Bartlem et al., 2015, Hahn et al., 2014). The presence of multiple risks increases the chance of chronic disease morbidity and mortality relative to one risk factor alone (Peters et al., 2019), and is a key contributor to the reduced life expectancy of up to 20 years experienced by people with a mental health condition compared to the general population (Campostrini et al., 2019, Chen et al., 2017, Firth et al., 2019).

Chronic disease risks are interrelated and measuring the prevalence of each, or multiple risks does not capture co-occurrence within individuals, nor allow exploration of risk patterns (McAloney et al., 2013). Cluster analysis can be used to examine clusters of risks that are likely to co-occur within individuals, enabling a more comprehensive understanding of risk patterns which may have important implications for the design of interventions. Further, exploration of associations between identified clusters and participant characteristics enables identification of groups who could most benefit from interventions. Most research that explores risk clustering has been undertaken with general population (Noble et al., 2015, Meader et al., 2016), vocational education students (Atorkey et al., 2021a), or child/adolescent samples (Whitaker et al., 2021). Among general population sample studies, cluster analysis has identified that tobacco smoking and harmful alcohol consumption cluster, as do inadequate nutrition and inadequate physical activity (Noble et al., 2015, Meader et al., 2016). Such data is valuable as it allows intervention approaches to consider the interactive nature of risks that are likely to cluster, rather than single-risk modification interventions (Prochaska, 2008, James et al., 2016). Studies suggest that interventions which simultaneously address clustered risks can potentially be more effective and cost-effective (Prochaska, 2008). Research also demonstrates that demographic factors such as lower socio-economic status and lower education tend to be associated with clusters characterised by a greater number of risks (Noble et al., 2015, Oftedal et al., 2019, Meader et al., 2016, Griffin et al., 2014, Paul et al., 2016).

To the authors’ knowledge, no studies have explored patterns of multiple risks using cluster analysis specifically among a sample of adults with mental health conditions. A small number of studies in general population samples have examined associations between cluster membership and presence of mental health variables (Griffin et al., 2014, Cao et al., 2020, Oftedal et al., 2019, Vermeulen-Smit et al., 2015, Ye et al., 2016, Jao et al., 2019), and have consistently found associations between membership of risk-dominant clusters and mental health condition presence or worse mental health symptoms (Oftedal et al., 2019, Jao et al., 2019, Ye et al., 2016, Vermeulen-Smit et al., 2015). For example, a large Australian study (n = 10,638) examined self-reported smoking status, diet quality, alcohol consumption, physical activity, and sleep quality, and identified four clusters: three of which were characterised by greater risk compared to one ‘healthy’ cluster (37% of sample) (Oftedal et al., 2019). Participants in each of the three risk-dominant clusters had significantly increased odds (OR = 2.14–6.02) of reporting frequent mental distress compared to participants in the ‘healthy’ cluster.

Given the identified higher prevalence of chronic disease and associated risks amongst people with a mental health condition, it is possible that the clustering of risks among this population may differ to that of the general population. However, no previous research has explored the clustering of risks specifically among a sample of people with a mental health condition. It is important to understand how, and to what extent chronic disease risks cluster among this population group, as the tendency for risks to aggregate and interact has important implications for preventive interventions and health policy. To address these evidence gaps, the aims of the current study were to conduct an exploratory analysis to: 1) identify clusters of key chronic disease risks (tobacco smoking, chronic alcohol consumption, acute alcohol consumption, fruit and vegetable intake, physical activity, strength activity, and body mass index (BMI) status) among a sample of people accessing community mental health services; and 2) explore possible associations between identified clusters and demographic characteristics and mental health conditions.

## 2 Methods

### 2.1 Design and setting

Cross-sectional surveys were undertaken with consumers of two government community mental health services in one Local Health District in a regional area of New South Wales (NSW), Australia. In Australia, government community mental health services are a major provider of mental health outpatient care (AIHW, 2020). The services provide specialised adult mental health care, that includes psychiatric rehabilitation, early diagnosis, and management to consumers with a range of mental health conditions (such as depression, anxiety, psychoses, bipolar disorder, eating disorders, comorbid substance-use) of varying severities.

Data were collected using Computer Assisted Telephone Interviews as part of a larger quasi-experimental controlled trial, involving consumers participating in two cross-sectional surveys: baseline in 2019 (April to October), and follow-up in 2020 (May to October). In the trial, one service received a practice change intervention and one continued to provide usual care. Included in the present study were all participants (from the control and intervention service) who completed a survey at baseline (prior to the intervention), and unique participants from the control service (i.e., not already identified in the baseline period) who completed a survey at follow-up (post intervention). The trial was approved by the Hunter New England Human Research Ethics Committee (Ref no. 18/11/21/4.06) and the University of Newcastle Human Research Ethics Committee (Ref no. H-2019–0108), and registered (ACTRN12619001379101).

### 2.2 Participants and data collection procedures

Adult consumers who had an in-person or telehealth appointment within the previous four months were identified via an electronic medical record system. Consumers were mailed a study information sheet that included a toll-free number to call if they did not wish to be contacted. Consumers were contacted approximately two weeks later via telephone by trained interviewers to assess further eligibility criteria (English speaking, mentally and physically capable of answering survey questions) and gain verbal consent to participate.

### 2.3 Measures

Survey items were based on previous surveys undertaken by the research team with mental health consumers (Bartlem et al., 2015, Fehily et al., 2020a, Fehily et al., 2020b) and validated items from recommended assessment tools (ABS, 2019):

#### 2.3.1 Risk factors

Participants were asked to self-report: how often they currently smoke cigarettes or any other type of tobacco product; their alcohol consumption using items from the AUDIT-C (Bush et al., 1998); the number of serves of fruit, and vegetables, typically consumed each day; physical activity level using the International Physical Activity Questionnaire-Short Form (Craig et al., 2003); and their current weight and height which was used to calculate BMI (WHO, 2000) (see Table 1).

**Table 1:** Health risk measures and definition of ‘at risk’ variables included in cluster analysis.

Risk Variable	Measures [*response options*]^c^	Definition of ‘at risk’
Tobacco smoking	How often they currently smoke cigarettes or any other type of tobacco product [*daily; at least once a week; less than once a week; not at all (quit<4 months ago); not at all (quit 4 months or more ago); never smoked*]	Smoked in the last four months, or quit within the last four months
Harmful chronic alcohol consumption	How often they consumed alcohol [*never, monthly or less, 2*–*4 times a month, 2*–*3 times a week, 4 + times a week*]; How many days per week they would have a drink containing alcohol [*0*–*7 days*]; How many standard drinks they would have on a typical drinking day [*1 or 2; 3 or 4; 5 or 6; 7 to 9; 10 +*]	>10 standard drinks/week
Harmful acute alcohol consumption	How often they would consume five or more standard drinks on one occasion [*never; less than monthly; monthly; weekly; daily or almost daily*]	>4 standard drinks/day on any day
Inadequate fruit & vegetable intake	Number of serves of fruit typically consumed each day [*0; 1; 2; 3; 4; 5; 6 or more*]; Number of serves of vegetables typically consumed each day [*0; 1; 2; 3; 4; 5; 6 or more*]	<2 serves/day of fruit and < 5 serves/day of veg
Inadequate moderate to vigorous physical activity	How many days, during the last seven days, they did *vigorous* physical activity (e.g. running, jogging, gym classes, boxing, soccer or squash) [*0*–*7 days*]; How many days, during the last seven days, they did *moderate* physical activity (e.g. fast walking, baseball, tennis, easy bicycling, volleyball, easy swimming), for at least ten minutes at a time [*0 –7 days*]; Participants who engaged in vigorous and/or moderate activity on at least one day were asked to report, respectively, how many minutes per session on average they spent doing these activities [*open numerical*]	<150 min moderate activity or, <75 min vigorous activity or, less than an equivalent combination of both^a^
Inadequate strength activity	Strength activity was measured by asking participants to report the number of days during the last week they engaged in any type of muscle strengthening activities (e.g. exercises using free weights, body weight exercises or gym-based strength exercises) [*0 –7 days*]	<2 days/week of including strength/resistance in physical activity
High BMI^b^	Current weight (kg or lbs) [*open numerical*]; Current height (cm or feet/inches) [*open numerical*]	BMI >= 25 (Overweight or Obese)

a an equivalent combination of moderate and vigorous activity was calculated by dividing the number of moderate activity minutes per week by two, then adding the number of vigorous activity minutes per week. If this total was<75, then considered inadequate (i.e. at risk).

b Overweight (25.0–29.9) and Obese (30.0 + ) BMI levels were classified as high BMI, representing the BMI risk variable. BMI calculated as weight in kilograms divided by height in metres squared) (WHO, 2000).

c all items included a ‘don’t know’ and ‘refused’ option.

#### 2.3.2 Demographic characteristics and mental health conditions

Gender, age, marital status, highest level of education achieved, employment status and identification as Aboriginal and/or Torres Strait Islander were assessed. Participants also reported the mental health condition/s they were receiving care for from the service (depression, anxiety, schizophrenia, bipolar disorder, Post Traumatic Stress Disorder (PTSD), personality disorder, substance use disorder and other).

### 2.4 Statistical analysis

Statistical analyses were conducted using SAS v9.4 (SAS Institute, Cary, North Carolina, USA); p < 0.05 (two-tailed) was used to indicate statistical significance. Consistent with previous research exploring clustering of multiple risks (Champion et al., 2018, Kwan et al., 2016, Oftedal et al., 2019), prevalence of each risk was represented by a categorical variable reflecting adherence to Australian National Guidelines (see Table 1) with seven risk variables calculated for the cluster analysis. Six dichotomous variables were calculated (at risk/non-adherence to guideline, vs not at risk/adherence to guideline): (1) tobacco smoking; (2) harmful chronic alcohol consumption; (3) harmful acute alcohol consumption; (4) inadequate fruit and vegetable intake; (5) inadequate physical activity; and (6) inadequate strength activity. Acute and chronic alcohol consumption, and inadequate physical activity and strength activity were considered separately based on the Australian national guidelines (Australian Government Department of Health, 2014, National Health and Medical Research Council, 2020) and their distinct impact on health and chronic conditions (Westcott, 2012, Churilla et al., 2012). For the seventh risk variable, BMI calculations were classified into four levels: underweight (<18.5); healthy weight (18.5–24.9); overweight (25.0–29.9), and obese (30.0+), where (7) high BMI was classified as overweight or obese (see Table 1). Behavioural risk variables were calculated as at risk if participants responded with ‘don’t know’ for any items in the variable calculation (Table 2 footnote j) (Bartlem et al., 2015, McElwaine et al., 2013, Tremain et al., 2017, Metse et al., 2017), and BMI was considered missing if participants responded to either height or weight items with’don’t know’. Descriptive statistics summarised demographic characteristics, mental health conditions, and risk variables.

**Table 2:** Demographic characteristics, mental health conditions and risks.

Variable	N (Total N = 567)	%
Gender		
Female	345	60.8
Male	220	38.8
Transgender or gender non-conforming	2	0.4
Age		
18 – 34	206	36.3
35 – 54	257	45.3
55+	104	18.3
Employment Status		
Employed^b^	136	24.0
Unemployed	120	21.2
Unable work due to health reasons	231	40.7
Other^c^	80	14.1
Marital Status		
Never married	285	50.3
Married or living together in a relationship	136	24.0
Other^d^	146	25.7
Education Level		
Some high school or less^e^	210	37.0
Completed high school certificate	92	16.2
Technical and further education (TAFE) certificate or diploma	212	37.4
Diploma, University degree or higher	53	9.3
Aboriginal or Torres Strait Islander	77^a^	13.6
Mental Health Condition^f^^g^		
Depression	343	60.5
Anxiety	302	53.3
Schizophrenia or other psychotic disorder	150	26.5
Bipolar disorder	119	21.0
Post-traumatic stress disorder	87	15.3
Personality disorder	83	14.6
Substance use disorder	36	6.3
Eating disorder	29	5.1
Obsessive-compulsive disorder	9	1.6
Other^h^	17	3.0
Behavioural Risk Variables i, j		
Tobacco smoking	303	53.5
Harmful chronic alcohol consumption	114	20.1
Harmful acute alcohol consumption	246	43.5
Inadequate fruit and vegetable intake^k^	355	66.0
Inadequate moderate to vigorous physical activity	428	75.5
Inadequate strength activity	464	81.8
BMI^i^		
Underweight (<18.5)	23	4.6
Healthy weight (18.5 – 24.9)	137	27.5
High BMI^l^ (25.0 + )	339	67.9

a n = 1 Torres Strait Islander.

b includes full time, part time, casual, on maternity leave.

c includes home duties, student, retired, other.

d includes separated, divorced, widowed.

e includes never attended school, some primary school, completed primary school, some high school, school certificate.

f participants could report one or more mental health conditions.

g N = 18 report did not report a mental health condition.

h other conditions reported included ADHD, Autism and Epilepsy.

i there was some variability in total n sizes for each variable (n BMI = 499; n fruit and vegetable intake = 538; n smoking = 566; n both alcohol = 566; and n both activity = 567) due to missing data.

j the proportion of participants at risk due to ‘‘don’t know’ responses, ranged from 0% (smoking) to 4.3% (chronic alcohol consumption).

k 88.5% (n = 476) were at risk for vegetable intake, and 66.5% (n = 358) were at risk for fruit intake. N = 29 not asked as had an eating disorder.

l 24.6% (n = 123) were overweight (25.0 – 29.9), and 43.3% (n = 216) were obese (30.0 + ).

The statistical approach used to examine clustering was latent class analysis (LCA), where participants were probabilistically allocated to latent clusters according to their pattern of the 7 risk variables. Latent Class models are parameterised according to 1) an assumed known number of classes, 2) the proportion of each class (class membership probabilities), and 3) the item response proportions within each class. The unknown parameters from this model were estimated using the Expectation-Maximisation algorithm in PROC LCA (Lanza et al., 2007). The number of latent classes was guided by comparing Akaike information criterion and Bayesian information criterion measures of fit between models with the number of latent classes varied between 2 and 10, as well as considering the interpretability of the resulting models. Participants were subsequently allocated a single class based on the highest-class membership probability produced for that individual; corresponding item-response probabilities were plotted accordingly to assist in the interpretation of the models. The item-response probability for risk ‘high BMI’ was calculated by adding the probability for the overweight level of BMI and the obese level of BMI.

Associations between best latent class membership and the following 13 participant characteristics were determined using separate univariate multinomial logistic regressions (age, gender, marital status, education, employment, Aboriginality, depression, anxiety, schizophrenia, bipolar disorder, personality disorder, PTSD, substance use disorder (yes/no for each mental health condition)). Chi-square odds ratios, 95% confidence intervals, number of observations used (N), pairwise p-values and overall Type III p-values were produced. Multivariable models were constructed using variables associated with the outcome (threshold p < 0.25) from corresponding univariate analyses; adjusted odds ratios, 95% confidence intervals, number of observations used (N), pairwise p-values and Type III p-values were presented with Cluster 1 as the reference category.

## 3 Results

### 3.1 Sample characteristics

**Fig. 1:**
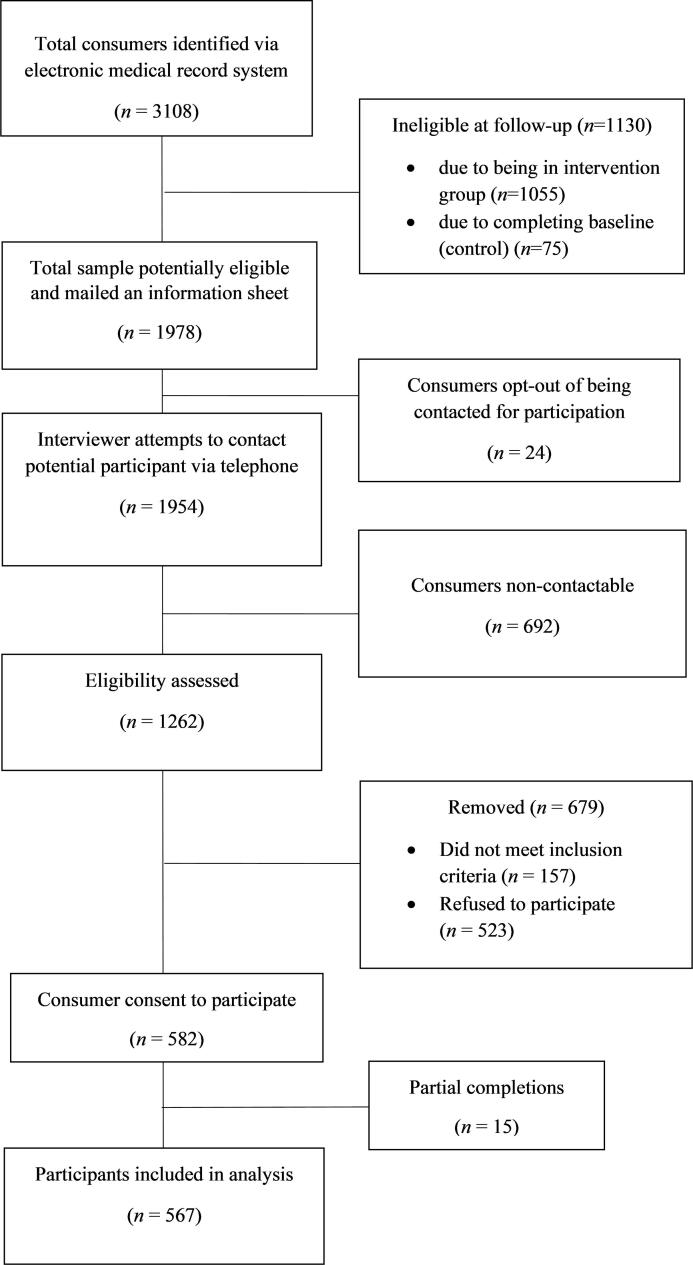
Flow diagram of participant recruitment.

Fig. 1 illustrates the participant recruitment process. Of 1105 eligible consumers, 567 participants completed the survey (61% female; mean age = 40.8 years). Table 2 outlines the prevalence of demographic characteristics, mental health conditions and risks for the sample.

### 3.2 Risk clusters

**Fig. 2:**
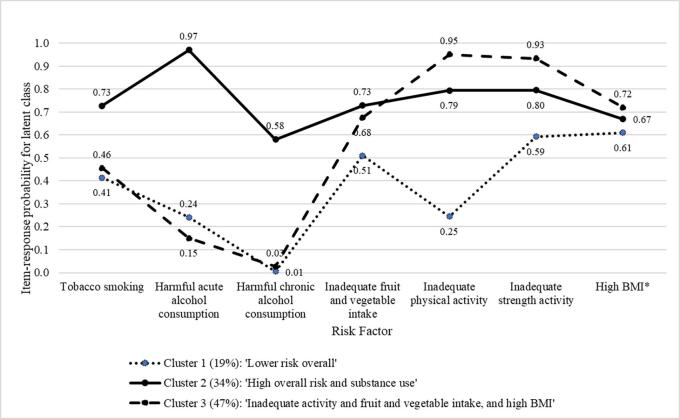
Item-response probabilities for risks by latent class. *Probabilities for BMI levels of overweight (C1: 0.28; C2: 0.31; C3: 0.19) and obesity (C1: 0.34; C2: 0.36; C3: 0.52) were added to reflect high BMI shown in the graph. See Supplementary material for individual item-response probabilities.

Three clusters were selected as appropriate based on the interpretability of the identified clusters in the LCA (see Fig. 2 which depicts the risk probability profiles of the clusters). Cluster 1 (19.1% of participants) was characterised by relatively lower risk overall (with four risks < 0.5 probability and three risks > 0.5 probability) whereas other clusters had higher probabilities for all risks, apart from harmful acute alcohol consumption in Cluster 3. Cluster 2 (34.0% of participants) was characterised by all risks (all risks > 0.5 probability): tobacco smoking (0.73); both harmful alcohol consumption variables (0.97 and 0.58 for acute and chronic, respectively); inadequate fruit and vegetable intake (0.73); both types of inadequate activity (0.79 and 0.80 for physical and strength, respectively); and high BMI (0.67). Cluster 3 (46.9% of participants) was characterised by inadequate fruit and vegetable intake (0.68), both types of inadequate activity (0.95 and 0.93 for physical and strength, respectively), and high BMI (0.72 probability).

### 3.3 Cluster associations with demographic characteristics and mental health conditions

Univariate associations of clusters with demographic characteristics and mental health conditions are reported in Table 3. The univariate analysis found that 8 variables had a p-value of < 0.25 (age, gender, martial, education, employment, depression, personality disorder, and substance use disorder) and these were entered into the multinomial logistic regression model. In the multivariable model (reference category: Cluster 1), gender (p < 0.0001), age (p = 0.0125), presence of a personality disorder (p = 0.0036), education level (p = 0.0446) and presence of a substance use disorder (p = 0.0381) were found to be significantly associated with latent cluster membership based on respective type III p-values (Table 3). When examining pairwise comparisons, participants with a university degree or higher education level had lower odds of Cluster 2 membership than Cluster 1 (OR: 0.308) relative to those with some high school or less (p = 0.0092). People with a substance use disorder had higher odds of Cluster 2 membership than Cluster 1 (OR = 11.953), relative to those without a substance use disorder (p = 0.0168). No other pairwise associations were significant for Cluster 1 versus Cluster 2 membership (p > 0.05). Females had higher odds of Cluster 3 membership than Cluster 1 (OR = 2.907), relative to males (type-III p < 0.0001). Those aged over 55 also had higher odds of Cluster 3 membership than Cluster 1 (OR = 3.180), relative to those aged 18 to 34 (p = 0.0061). People who were unable to work due to health reasons had higher odds of Cluster 3 membership compared to Cluster 1 (OR = 2.766), relative to employed individuals (p = 0.0028), however interpretations here are cautious as the overall effect of employment on cluster membership (type III) was not significant. Participants with a personality disorder had lower odds of Cluster 3 membership than Cluster 1 (OR = 0.365), compared to those without a personality disorder (p = 0.0062). Those with a university degree or higher education level also had lower odds of Cluster 3 membership than Cluster 1 (OR = 0.392), relative to those who completed some high school or less (p = 0.0227).

**Table 3:** Estimates of multivariable multinomial logistic regression model of latent cluster allocation (modelling probability of being in Cluster 1).

Variable	Characteristic	Cluster 2		Cluster 3		Type III P-Value
		*Odds Ratio (95% CI)*	*Pairwise P-Value*	*Odds Ratio (95% CI)*	*Pairwise P-Value*	
Age	35–54 vs. 18–34	1.273 (0.720, 2.251)	0.4059	1.749 (0.995, 3.073)	0.0519	0.0125*
	55 + vs. 18–34	1.229 (0.509, 2.970)	0.6461	3.180 (1.392, 7.264)	0.0061*	
Gender^a^	Female vs. Male	1.381 (0.815, 2.338)	0.2303	2.907 (1.727, 4.891)	<0.0001*	<0.0001*
Marital	Married or living together in a relationship vs. Never married	1.300 (0.687, 2.460)	0.4199	1.544 (0.832, 2.862)	0.1683	0.1711
	Other vs. Never married	1.749 (0.881, 3.471)	0.1100	1.146 (0.586, 2.241)	0.6899	
Education	Completed HSC vs. Some high school or less	1.492 (0.687, 3.239)	0.3114	1.590 (0.734, 3.444)	0.2394	0.0446*
	TAFE certificate or diploma vs. Some high school or less	0.952 (0.534, 1.700)	0.8692	0.815 (0.461, 1.440)	0.4804	
	University degree or higher vs. Some high school or less	0.308 (0.127, 0.747)	0.0092*	0.392 (0.175, 0.877)	0.0227*	
Employment	Other vs. Employed	1.142 (0.524, 2.488)	0.7385	1.160 (0.535, 2.514)	0.7074	0.0572
	Unable to work due to health reasons vs. Employed	1.518 (0.769, 2.993)	0.2287	2.766 (1.419, 5.391)	0.0028*	
	Unemployed vs. Employed	1.293 (0.643, 2.600)	0.4710	1.528 (0.758, 3.079)	0.2360	
Depression	Yes vs. No	0.987 (0.593, 1.643)	0.9595	1.536 (0.930, 2.536)	0.0933	0.0703
Personality disorder	Yes vs. No	0.874 (0.437, 1.747)	0.7025	0.365 (0.177, 0.751)	0.0062*	0.0036*
Substance use disorder	Yes vs. No	11.953 (1.563, 91.423)	0.0168*	7.504 (0.954, 59.014)	0.0555	0.0381*

*Asterix denotes (P=<0.05).

a Gender included male and female, where transgender or gender non-conforming was excluded from the respective logistic regression model due to low numbers (n = 2).

## 4 Discussion

This study was the first to use cluster analysis to explore patterns of modifiable chronic disease risks among people accessing community mental health services. Three risk clusters were identified: Cluster 1, ‘lower risk overall’, exhibiting the lowest risk of the three clusters; and Cluster 2 and 3, ‘high overall risk and substance use’ and ‘inadequate activity and fruit and vegetable intake, and high BMI’, respectively, exhibiting higher risk. Four demographic variables and two mental health conditions were associated with the risk-dominant clusters when compared to the lower risk cluster.

### 4.1 Clusters

When compared to other clusters within this study, Cluster 1 was characterised by the relatively lowest probabilities of most risks, Cluster 2 was characterised by the relatively highest probabilities of tobacco smoking, both types of alcohol consumption and inadequate fruit and vegetable intake, and Cluster 3 was characterised by the relatively highest probabilities of both types of inadequate activity, inadequate fruit and vegetable intake, and high BMI. Prior research in general population samples has typically identified three to four clusters, of which one is ‘healthy’ (Noble et al., 2015, Vermeulen-Smit et al., 2015, Pettigrew et al., 2021, Jao et al., 2019, Cao et al., 2020, Di Benedetto et al., 2019, Oftedal et al., 2019), and one cluster characterised by elevated substance use (Noble et al., 2015, Jao et al., 2019, Vermeulen-Smit et al., 2015, Griffin et al., 2014, Noel et al., 2013, Funderburk et al., 2008).

The profile and proportion of Cluster 1 differs considerably to that typically found in general population research. Although identified as the ‘healthier’ cluster, Cluster 1 nevertheless evidenced considerable levels of risk with > 0.5 probability of three risks and almost 0.5 probability of tobacco smoking. In comparison, previous studies using cluster analysis in general population samples have reported ‘healthy’ clusters characterised by zero or one risks (Meader et al., 2016, Noble et al., 2015, Whitaker et al., 2021), and the probability of tobacco smoking is typically < 0.1 in such clusters (Hutchesson et al., 2021, Oftedal et al., 2019, Pettigrew et al., 2021). Additionally, studies in the Netherlands and Australia with general population and University samples report ‘healthy’ clusters to account for the largest proportion (54–80%) of the sample (Vermeulen-Smit et al., 2015, Oftedal et al., 2019, Di Benedetto et al., 2019). In contrast, the ‘healthy’ cluster (Cluster 1) accounts for the smallest proportion (19.05%) relative to Cluster 2 (34.04%) and Cluster 3 (46.91%). Although direct comparisons are not possible due to the lack of a comparison group, these findings provide support for existing evidence that multiple chronic disease risks are likely more prevalent among people with a mental health condition compared to the general population. Further research including a non-mental health comparison group would strengthen this finding. Regardless, these results reinforce the need for action to address multiple risks for this population.

A large proportion of the sample were allocated to clusters characterised by higher risk. Cluster 2 was distinguished based on the probabilities of substance-related risks, yet demonstrated > 0.5 probabilities for all risks. The current finding suggest people with a mental health condition engaging in substance-related risks are likely to also have inadequate physical and strength activity levels as well as inadequate fruit and vegetable consumption. Cluster 3 accounted for almost half of participants and had > 0.5 probabilities for both types of inadequate activity, inadequate fruit and vegetable intake, and high BMI. Clustering of such risks is supported by previous research suggesting that physical activity impacts diet and weight (Beaulieu et al., 2016, Fenton et al., 2021, Höchsmann et al., 2020), a potentially more salient association for people with a mental health condition due to health promoting barriers such as medication induced cravings and low motivation (Bailey et al., 2018, Scott and Happell, 2011). The probability of tobacco smoking in Cluster 3 (0.46) is typically higher than previous studies which have identified ‘unhealthy’ clusters that are not characterised by smoking (probabilities ranging from 0.04 to 0.29) (Atorkey et al., 2021b, Hutchesson et al., 2021, Oftedal et al., 2019, Pettigrew et al., 2021), evidence of tobacco smoking being a particularly prevalent risk among this population group, likely due to the perceived role of tobacco smoking in mental illness symptom management (Bailey et al., 2018, Keller-Hamilton et al., 2019).

Although it is difficult to make direct comparisons with previous studies due to various population groups and measurement approaches (such as different tools to measure risk and different combinations of risks included), the current study provides evidence of clustering of addictive behaviours requiring restraint or abstinence (smoking and alcohol in Cluster 2) and health promoting behaviours requiring active engagement (physical activity and fruit and vegetable consumption in Cluster 3) (de Vries et al., 2008, Noble et al., 2015). The clustering patterns identified, and the large proportion of participants (81%) in the risk-dominant clusters, highlights the need to target multiple risks. Despite conflicting points of view regarding multi-risk interventions, there is evidence to suggest that interventions targeting clustered risks may be more effective in aiding overall behaviour change than singular risk interventions (Cradock et al., 2017, Nigg and Long, 2012, Sweet and Fortier, 2010) as well as being less costly (Park et al., 2013, Fehily et al., 2020a, Fehily et al., 2020b, Ritzwoller et al., 2011), potentially due to the synergistic effects that change in one risk behaviour has on another (Hammami et al., 2020, Huang et al., 2012, Spring et al., 2012). However, to the authors’ knowledge, no studies have directly compared singular and multi-risk interventions among people with a mental health condition. Finding ways to develop and test multi-risk interventions for people with a mental health condition to understand the most acceptable and effective approaches to addressing multiple risks is warranted.

### 4.2 Associations

This study identified that people accessing community mental health services with higher education levels were less likely to be allocated the risk-dominant clusters, which is consistent with general population research (Noble et al., 2015, Oftedal et al., 2019, Pettigrew et al., 2021, Griffin et al., 2014, Hsu et al., 2013). Additionally, females and older people were more likely to be in Cluster 3 than Cluster 1, compared to males and those aged 18–34, respectively. As Cluster 3 exhibited the relatively lowest probability for harmful acute alcohol consumption and relatively highest probabilities for both types of inadequate activity, these findings are consistent with previous research demonstrating females and older people are less likely to binge drink (Pettigrew et al., 2021, Atorkey et al., 2021a, Atorkey et al., 2021b, Shaw and Agahi, 2012, Lee et al., 2012, Griffin et al., 2014, Paul et al., 2016, Noel et al., 2013) as well as engage in adequate physical activity (Alley et al., 2017, Bennie et al., 2016) than males and younger people, respectively. Understanding characteristics associated with certain clusters may provide additional considerations for tailoring multi-risk interventions.

The current sample included a broad range of mental health diagnoses, with only two conditions demonstrating significant associations. It is not surprising that substance use disorder had increased odds of Cluster 2 membership, given previously identified associations between substance use disorder, tobacco smoking, and alcohol consumption (Weinberger et al., 2017, Weinberger et al., 2016, Kelly et al., 2012). The finding that personality disorder had significantly lower odds of Cluster 3 membership is difficult to interpret, and no previous studies have assessed such an association. Future research should test associations between risk clusters and specific mental health conditions with larger sample sizes to allow stronger conclusions to be made. The number of participants with particular mental health conditions was limited (see Table 2), and therefore assessing associations with specific conditions was exploratory. Notwithstanding, findings of minimal differences across diagnoses are consistent with research suggesting that people with any mental health condition (i.e. not limited to depression or severe mental illness) experience high levels of chronic disease risk. Taken with previous meta-analyses demonstrating a life expectancy gap for all mental health conditions (Walker et al., 2015), the findings reinforce the importance of interventions focused on the prevention of chronic disease risks to be provided systematically and routinely to all people accessing community mental health services, in line with national and state health service guidelines (Australian Health Ministers’ Advisory Council, 2017, NSW Ministry of Health, 2021), and international recommendations (WHO, 2021).

### 4.3 Limitations

Some further study limitations should be acknowledged. Firstly, participants were people accessing community mental health services within NSW, Australia. It is unclear how generalisable the results are to people accessing other mental health care settings, or to people with a mental health condition not currently receiving care. Secondly, the data is self-report and therefore may under report risk as studies have found that the prevalence of risk factors in the community are higher according to gold standard data sources compared to self-report data (Althubaiti, 2016, Cronin et al., 2009, Newell et al., 1999, Prince et al., 2020, Spitzer and Weber, 2019). Additionally, some data collection coincided with the COVID-19 lockdowns (^∼^25%), the first experienced in this area of Australia. Research exploring the impact of COVID-19 lockdowns in Australia found the general population were more likely to engage in risks than before the lockdowns (Stanton et al., 2020, Brindal et al., 2021, Glenister et al., 2021, Arora and Grey, 2020, Phillipou et al., 2020). To the authors’ knowledge no research has been conducted with samples of people with a mental health condition, and whilst participants were prompted to respond with how they would normally engage in behaviours, it is unclear how the lockdowns impacted on reporting of risks. Further, characteristics of mental health conditions, such as severity and medications, may have an impact on risks however were not measured. Finally, due to the cross-sectional nature of the study, it is not possible to draw conclusions regarding the causality of the relationship between the clusters and mental health conditions examined.

## 5 Conclusion

Findings of this study strengthen previous research demonstrating people with a mental health condition have a high likelihood of engaging in multiple chronic disease risks, and adds a perspective of risk clustering. Minimal associations between clusters and participant characteristics suggest that regardless of demographics and diagnoses, this issue is important across all people with a mental health condition. Consistent with evidence and international health guidelines (World Health Organization, 2021, Kluge et al., 2020), the current findings can assist with planning intervention approaches to target multiple risk factors which commonly occur together and what factors to consider to adapt interventions accordingly. Further research could explore the effectiveness of multi-risk interventions and involve consumers and other stakeholders in their development (NSW Ministry of Health, 2018, Mental Health Commission of New South Wales, 2014). Understanding the most effective approaches to addressing the high prevalence and clustering of risks among people with a mental health condition may be an important step to redressing the physical health and life expectancy inequalities experienced.

## CRediT authorship contribution statement

**Casey Regan:** Conceptualization, Methodology, Writing – original draft, Writing – review & editing, Project administration, Funding acquisition. **Caitlin Fehily:** Conceptualization, Methodology, Writing – review & editing, Supervision. **Elizabeth Campbell:** Conceptualization, Methodology, Writing – review & editing, Supervision. **Jenny Bowman:** Conceptualization, Methodology, Writing – review & editing, Supervision, Funding acquisition. **Jack Faulkner:** Conceptualization, Methodology, Formal analysis, Writing – review & editing. **Christopher Oldmeadow:** Conceptualization, Methodology, Formal analysis, Writing – review & editing. **Kate Bartlem:** Conceptualization, Methodology, Writing – review & editing, Supervision, Funding acquisition.

## Declaration of Competing Interest

The authors declare that they have no known competing financial interests or personal relationships that could have appeared to influence the work reported in this paper.
