# Supplementary material for: Clustering of chronic disease risks among people accessing community mental health services
Source: Prev Med Rep. 2022 Jun 27;28:101870. doi: 10.1016/j.pmedr.2022.101870 (PMC9256721; doi:10.1016/j.pmedr.2022.101870)
Supplement: Supplementary data 1 [file mmc1.docx]

# **Supplementary Material**

| **Table 1. Latent class analysis: Probability of having each risk for each class** | | | |
| --- | --- | --- | --- |
|  | Cluster 1 (19%) | Cluster 2 (34%) | Cluster 3 (47%) |
| Tobacco smoking | 0.41 | 0.73 | 0.46 |
| Harmful acute alcohol consumption | 0.24 | 0.97 | 0.15 |
| Harmful chronic alcohol consumption | 0.01 | 0.58 | 0.03 |
| Inadequate fruit and vegetable intake | 0.51 | 0.73 | 0.68 |
| Inadequate physical activity | 0.25 | 0.79 | 0.95 |
| Inadequate strength activity | 0.59 | 0.80 | 0.93 |
| Overweight | 0.28 | 0.31 | 0.19 |
| Obesity | 0.34 | 0.36 | 0.52 |
